# Veterans with Service, Emotional Support, and Companion Dogs: Examining the Relationship Between Demographics, Health Characteristics, and Intensity of Human–Dog Relationships

**DOI:** 10.3390/bs16010016

**Published:** 2025-12-21

**Authors:** Cheryl A. Krause-Parello, Christine Spadola, Jacquelyn Baldwin, Joy Sessa, Erika Friedmann

**Affiliations:** 1 Division of Research, Florida Atlantic University, Boca Raton, FL 33431, USA; 2 School of Social Work, University of Texas at Arlington, Arlington, TX 76019, USA; christine.spadola@uta.edu; 3 Institute for Human Health and Disease Intervention, Florida Atlantic University, Boca Raton, FL 33431, USA; jbaldwin2018@health.fau.edu (J.B.); joysessa@gmail.com (J.S.); 4 School of Nursing, University of Maryland, Baltimore, MD 21201, USA; friedmann@umaryland.edu

**Keywords:** veterans, PTSD, service dogs, emotional support dogs, companion dogs, mental health, nonpharmacological treatment, human–animal relationship, human–dog relationship, veteran well-being

## Abstract

Dog ownership may be an effective nonpharmacological, rehabilitative approach to improve veterans’ mental health and well-being. For three functional types of dogs—service, emotional support, and companion—little is known about the demographic and health characteristics of veterans and the dog types they own. This study examined veteran demographics and health characteristics stratified by functional dog type and intensity of the relationship. A cross-sectional online survey with several reliable/valid health and well-being instruments was administered to veterans with a service, emotional support, or companion dog. A convenience sample of veterans (*N* = 242) with a mean age of 46.9 (*SD* = 13.4) participated in this study. There were 143 males, 95 females, and 2 participants with another identity. The majority were white (71%). The Army (48.3%) was the most represented branch. Significant differences were found between veteran health characteristics [suicidal ideation, PTSD, anxiety, and physical well-being based on the functional dog type owned]. Service dog owners had a significantly more intense relationship with their dog. This study provides insight into the role dogs may play in improving mental health and well-being in veterans. To prevent further disability in veterans, clinicians should consider incorporating the right functional dog type in personalized care plans.

## 1. Introduction

United States (U.S.) military veterans comprise about 7 percent of the U.S. population and are at risk of disabling mental health challenges, including post-traumatic stress disorder (PTSD), anxiety, depression, and loneliness (Vespa, 2020). Human–animal interaction has been credited with health benefits for individuals across the spectrum (Cherniack & Cherniack, 2014; Friedmann et al., 2015). Dogs can enhance human social connectedness by facilitating social interaction with others (Wells, 2004). Empirical evidence has demonstrated that pet ownership reduces stress, isolation, and loneliness across the human lifespan (Carr et al., 2021; Kertes et al., 2017; Taniguchi et al., 2018). Other studies have shown that dog ownership and bonding can decrease PTSD symptoms and self-judgment while increasing self-compassion (Bergen-Cico et al., 2018). Simple interactions, such as taking a walk with a dog, have demonstrated promise in decreasing stress as well as PTSD symptom severity (Krause-Parello et al., 2020). Dogs benefit from their relationship with humans too, enhancing physical activity and play (Müllersdorf et al., 2009).

Veterans who interact with dogs gain therapeutic health benefits (Krause-Parello et al., 2019; Krause-Parello et al., 2016a). Three functional dog types, including service dogs, emotional support dogs, and companion dogs (pets), have been identified as providing a complementary and rehabilitative approach to improve veterans’ mental health and well-being (Krause-Parello et al., 2021). Service dogs (SDs) are defined as working dogs specially trained to respond to particular needs of their owners, such as physical and mental health needs (Krause-Parello et al., 2016b). Psychiatric service dogs perform tasks such as deep pressure therapy, hypervigilance reduction, and tactile stimulation, among others. Reported benefits of service dogs within the veteran population include decreased PTSD symptom severity, depression, and anxiety (Leighton et al., 2024). Additional benefits reported include higher quality of life and higher social functioning (O’Haire & Rodriguez, 2018). A study assessing female veterans before and after an 8-week service dog training program showed decreased PTSD symptom severity, anxiety, and perceived stress for veterans after the training program (Krause-Parello et al., 2025). These functional dogs are permitted in public areas (Taylor et al., 2015) under the Americans with Disabilities Act (ADA); for more information about protections provided by the ADA, visit the ADA website (U.S. Department of Justice, n.d.).

Emotional support dogs (ESDs) are dogs that may be trained or untrained (natural behaviors) to provide comfort and support in lessening the discomfort associated with their owner’s psychiatric needs (Krause-Parello et al., 2016b). Their primary role is to provide emotional or psychological support through their presence and relationship with the human rather than from performing specific tasks (Ferrell & Crowley, 2021). While they do receive housing accommodation, this functional dog type does not possess the same range of protections as SDs under the ADA (Krause-Parello et al., 2016b). Reported benefits of emotional support dogs include decreased depression, anxiety, and loneliness (Hoy-Gerlach et al., 2021). In addition, they have been reported to improve mood (Thelwell, 2019).

Companion dogs (CDs) are commonly referred to as a personal pet. This functional dog type includes dogs that are not trained to provide support for those with psychiatric needs (Krause-Parello et al., 2016b). Companion dogs do not have public access rights and do not fall under the scope of protections provided by the ADA (Krause-Parello et al., 2016b). Reported benefits of companion dogs within the veteran population include reduced loneliness and increased social interactions and emotional well-being (Seawell et al., 2024). Additional benefits reported include improved physical activity, responsibility, companionship, and stress reduction (Kruger et al., 2014).

Although all three dog types are credited with improving veterans’ health, the distinction in function has not been investigated. The impact of the three functional types of dogs on veterans’ health may depend on veteran demographics and health characteristics and the intensity of the veteran–dog relationship. Studies to date that examine veteran health and wellness often include only one or two subsets of dog types (e.g., service dogs only); therefore, it is valid and necessary to conduct a study comparing the three functional dog types: SD, ESD, and CD.

Building on the evidence outlined above and guided by the biopsychosocial theoretical framework, the purpose of this study is to describe the relationship of veteran demographics and health characteristics, dog functional type, and intensity of the human–dog relationship, filling a fundamental gap in the literature.

### Research Question

What is the relationship between veteran demographics and health characteristics, dog functional type, and the intensity of the human–dog relationship?

To answer this question, we examine the following points:

(1) The differences in demographic and health characteristics among veterans who cohabitate with an SD, ESD or CD.
(2) The differences in the intensity of the veteran’s relationship with their dog based on the dog’s functional type.
(3) The relationships between veteran health outcomes and veterans’ relationship with their dogs in the entire group and in each of the dog functional types.

## 2. Methods

### 2.1. Theoretical Framework

This study is informed by the biopsychosocial theoretical framework, an integrated, holistic approach to health and wellness that considers biological, psychological, and social factors (Suls & Rothman, 2004). The validated assessments that were employed represent all three bio-psycho-social domains to gain a multi-dimensional account of veteran health and wellness. Veteran health involves integration of biological, psychological, and social components, which interact with each other. Changes in any component affect other constructs within that component as well as constructs within the other components. Changes can either promote or inhibit overall health/wellness through inter-related biological, psychological, and social mechanisms. In the biopsychosocial model, dogs can be conceptualized as a form of social support that would impact other aspects of social health, as well as biological and psychological health (Krause-Parello, 2012). Therefore, the variables in this study were chosen to reflect the biological, social, and psychological components of the theoretical framework.

### 2.2. Study Design

This study used a cross-sectional online survey design.

### 2.3. Ethical Oversight

This study was approved and deemed exempt by Florida Atlantic University’s institutional review board (IRB) (IRB number 1773112).

### 2.4. Sample and Setting

To be included in the study, participants had to meet the following criteria: a U.S. military veteran, able to read and write English, aged 18 years or over, and currently own one of the following functional dog types: SD, ESD, or CD. Participants completed the surveys online in a location of their choice.

### 2.5. Recruitment

Recruitment strategies included a social media campaign via designated Facebook, Instagram, and Twitter accounts and advertisement through non-profit, veteran-serving agencies.

### 2.6. Study Instruments

A Demographics and Characteristics Questionnaire was developed specifically for this study. Items included veteran demographics such as sex, education, age, ethnicity, race, marital status, living arrangements, and military-related questions (e.g., rank, branch, combat exposure, years of service). Dog demographics included age, sex, breed, type of dog (SD, ESD, or CD), and length of dog ownership.

Post-Traumatic Stress Disorder symptoms were measured using the PTSD Checklist for DSM-5 (PCL-5). The PCL-5 is a validated tool that assesses the 20 DSM-5 symptoms of PTSD and is widely used in research involving U.S. military veterans. The PCL-5 is a 20-item 5-point Likert-type scale with anchors ranging from 0 (not at all) to 4 (extremely high (Blevins et al., 2015). Total scores range from 0 to 80, with a higher score indicating a higher likelihood of PTSD. Excellent internal consistency was shown in a study with 140 veterans, with Cronbach’s alpha being 0.96 (Bovin et al., 2016). Cronbach’s alpha in this study was 0.97.

Anxiety was measured by the Patient Reported Outcomes Measurement Information System (PROMIS) Short Form (SF) v1.0—Anxiety 4a item bank (Pilkonis et al., 2011). This 4-item Likert-type scale uses anchors that range from 1 (never) to 5 (always). Total scores range from 4 to 20, with higher scores indicating higher levels of anxiety. In the development of the short form, excellent internal consistency was shown with the coefficient alpha being 0.93 (Pilkonis et al., 2011). Cronbach’s alpha in this study was 0.90.

Positive affect was measured by the PROMIS SF v1.0—Positive Affect 15a item bank (Salsman et al., 2014). It measures positive affective experiences (e.g., feelings of happiness, joy, and peace). This 15-item Likert-type instrument uses anchors ranging from 1 (not at all) to 5 (very much). Total scores range from 15 to 75, with a higher score indicating a more positive effect. In testing the item banks for psychological well-being, all 34 items in the full item bank for positive affect (PROMIS Bank v1.0—Positive Affect), which contains the items from the short form, exhibited reliabilities of >0.95 (Salsman et al., 2014). Cronbach’s alpha in this study was 0.97.

Depression was measured using the 8-item Patient Health Questionnaire Depression Scale (PHQ-8) (Kroenke et al., 2009). This Likert-type scale uses anchors that range from 0 (not at all) to 3 (nearly every day). Total scores range from 0 to 24, with a total score of 0 to 4 indicating no significant depressive symptoms, 5 to 9 indicating mild symptoms, 10 to 14 indicating moderate symptoms, 15 to 19 indicating moderately severe symptoms, and 20 to 24 indicating severe depressive symptoms. A coefficient alpha of 0.95 was reported in a study of 350 male U.S. military veterans with ages ranging from 41 to 89 (Silberbogen et al., 2013). Cronbach’s alpha in this study was 0.89.

Loneliness was measured using the UCLA 8 Loneliness Scale (Hays & DiMatteo, 1987). This 8-item Likert-type scale uses anchors that range from 1 (never) to 4 (always). Total scores range from 8 to 32, with a higher score indicating a higher degree of loneliness. Good internal consistency (*α* = 0.86) was reported for this scale from a study with 87 participants at least 65 years of age (Barron et al., 1994). Cronbach’s alpha in this study was 0.85.

Perceived Stress was measured using the 10-item Perceived Stress Scale (PSS-10) (Cohen et al., 1983). It measures the extent to which circumstances in one’s life are considered stressful. The PSS-10 is a Likert-type scale with anchors ranging from 0 (never) to 4 (very often). Total scores range from 0 to 40, with higher scores indicating higher perceived stress. A coefficient alpha of 0.82 was reported in a study of 768 adults over the age of 70 (Ezzati et al., 2014). Cronbach’s alpha in this study was 0.70.

Veterans’ perceptions of general physical and mental well-being were measured using the Veterans RAND 12 Item Health Survey (VR-12). This survey was developed from the Veterans RAND 36 Item Health Survey (VR-36), which was developed from the MOS RAND SF-36 Version 1.0 (Hays et al., 1993; Kazis et al., 2004, 2006a, 2006b; Rogers et al., 2004; Spiro et al., 2004). VR-12 is a Likert-type assessment with anchors varying throughout the 12 items. The 12 items are summarized into two separate scores for physical and mental health, a physical component score (PCS) and a mental component score (MCS). In this study, Cronbach’s alpha for the PCS was 0.43, and Cronbach’s alpha for the MSC was 0.32.

Suicidal ideation was measured using the Suicidal Ideation Attributes Scale (SIDAS) (Van Spijker et al., 2014). The 5-item Likert-type scale uses anchors ranging from 0 to 10. These 5 items measure frequency of suicidal thoughts, how much control respondents had over these thoughts, closeness to suicide attempt, distress caused by these thoughts, and the interference these thoughts had with daily activities over the past month (Van Spijker et al., 2014). Participants who answered 0 (Never) to the first item assessing frequency of suicidal thoughts did not continue on to answer the remaining 4 items. Total scores ranged from 0 to 50, with a higher score signifying greater suicidal ideation severity. Strong internal reliability (*α* = 0.91) was reported for this scale from an online study with 1352 adults (Van Spijker et al., 2014). Cronbach’s alpha in the current study was 0.85.

Companionship was measured using the PROMIS SF v2.0—Companionship 4a item bank (Hahn et al., 2014). It measures the perceived accessibility of someone with whom the participant can enjoy social activities. This 4-item Likert-type measure uses anchors ranging from 1 (never) to 5 (always). Total scores range from 4 to 20, with a higher score indicating higher perceived companionship. In a study with 141 post-9/11 military members and veterans with PTSD, the internal consistency for the short form was 0.93 (O’Haire & Rodriguez, 2018). Cronbach’s alpha in this study was 0.95.

Dog–owner relationships were measured using the Cat/Dog–Owner Relationship Scale (CDORS), a 32-item, validated tool designed to measure three primary components of human–dog and cat relationships: human/animal interaction, human/animal closeness, and perceived financial costs of animal ownership (Riggio et al., 2021). Each item is scored on a 5-point Likert scale. Coefficient alpha scales provide adequate reliability of the three subscales: interactions *α* = 0.84, emotional closeness *α* = 0.85, and cost *α* = 0.71 (Riggio et al., 2021). Cronbach’s alpha in this study was 0.89 for the total scale subscales: pet closeness had a Cronbach’s alpha of 0.86; perceived financial cost had a Cronbach’s alpha of 0.73; and pet interaction had a Cronbach’s alpha of 0.82.

### 2.7. Study Procedures

Data was collected from participants utilizing a Research Electronic Data Capture (REDCap) online survey. Veterans were able to access the survey via a customized link or by scanning the QR code provided by REDCap, both of which were on the flier used for recruitment. Once the veteran clicked on the link or scanned the QR code, they were brought to the REDCap webpage containing pre-screen questions and bot-identifying questions to ensure they met the inclusion criteria for the study before proceeding. If an answer given revealed the veteran was not eligible, they were told they did not meet the eligibility criteria, and the survey ended. If the veteran met all eligibility criteria, they were then brought to the consent form. Due to sensitive subjects being addressed in the survey, the following resources were listed on the consent form for veterans should they become distressed: contacting their primary care provider, the National Suicide Prevention Lifeline phone number, and the Veterans Crisis Line. Once the veteran read the consent form, provided their signature, phone number, email, dog’s name, dog’s functional type, and a photo of their dog, they were able to proceed to the sociodemographic questions and the other survey instruments listed in the previous section. Once one survey instrument was submitted, the participant was automatically brought to the next instrument until the survey was completed. If the participant only partially completed the survey, a member of the research team sent them an individualized link to the partial completion point to allow them the opportunity to finish. The photo of their dog was required to authenticate dog ownership and to be used in a gentle email reminder to complete the surveys. The participant would receive an automated email with their dog’s picture on top and a message that says “Hello! How are you and [dog’s name] doing?” The survey took about 20–35 min to complete, on average. As a form of appreciation, veterans received an electronic gift card from a national retailer following the completion of the surveys. They were given gift cards after completing the survey at each time point: $25 at time 1, $30 at time 2, and $45 at time 3. All surveys were confirmed, and the responses were validated prior to issuing gift cards.

### 2.8. Method for Data Analysis

Prior to data analysis, the data was cleaned. Analysis of data missingness revealed no significant concerns. Assumptions of each analytic technique were confirmed.

After completing descriptive statistics for all participants, the demographic and psychological health characteristics of the veterans and of the dogs in the three functional dog types were compared. ANOVAs were used for continuous variables and chi-squares were used for categorical variables. Bonferroni sequential corrections were used for multiple comparisons. Similar analyses were conducted to examine differences in veterans’ perceptions of the intensity of the relationships with their dogs for the entire CDORS and the three CDORS subscales. Correlations were used to examine the relationship of the health outcomes with the veterans’ relationships with their dogs in the entire group and in each of the dog functional types.

## 3. Results

The final sample comprised 242 participants; 81 participants had an SD, 80 had an ESD, and 81 had a CD.

### 3.1. Demographics of Veteran Participants

The veterans’ (*N* = 242) ages ranged from 24 to 78 years (*M* = 46.9, *SD* = 13.4; see Table 1). Participants were mostly male (59.6%) and white (71%). More than half of the respondents (55.4%) had a college bachelor’s degree or more education; 58.1% were married, and 65% owned the homes they lived in. Most participants were either employed full-time (36.4%), retired (18.2%) or disabled (27.3%). Seventy-five percent of the participants owned a dog as a child. Military branches represented included Army (48.3%), Air Force (19.4%), Navy (16.9%), Marines (14.0%), National Guard (7.0%), and Coast Guard (2.9%). A majority of the participants (54.4%) had not been exposed to combat during their military service.

### 3.2. Demographics of the Dogs

**Figure 1:**
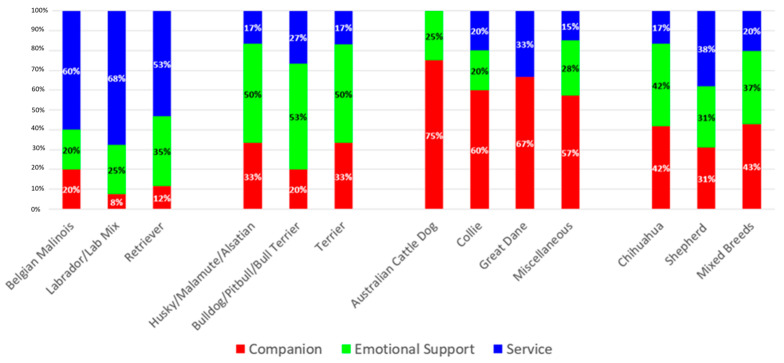
Percentage of Functional Dog Types by Breed.

The average age of the dogs was 5.51 years (*M* = 5.51, *SD* = 3.61); 97 were female (40%), and 141 were male (58%). Dogs’ ages differed among the three functional dog types [*F*(2,222) = 4.46, *p* = 0.013]. Companion dogs were significantly older than emotional support (*p* = 0.031) and service dogs (*p* = 0.030) (see Table 1). Sixteen breeds were represented by at least 2 dogs. The additional dogs were mixed breeds or other breeds. When dog breeds were categorized according to the percent of the breed in each functional type, three breeds were primarily (≥50%) SDs: Belgian Malinois, Labrador/lab mix, and Retrievers; 3 breeds were primarily ESDs: Husky/Malamute/Alsatian, Bulldog/Pitbull/Bull Terrier, and Terrier; 4 breeds were primarily CDs: Australian Cattle Dog, Collie, Great Dane, and other breeds; and 3 breeds had a more varied distribution of functional types: Chihuahua, Shepherd, and Mixed Breeds (see Figure 1).

### 3.3. Demographic and Health Characteristic Differences Among SDs, ESDs, and CDs

The veterans with SD, ESD, and CDs were similar in demographics and military characteristics, with the exception of combat exposure [*Chi sq* (2) = 11.63, *p* = 0.003] (Table 1). Although 45.6% of the sample reported combat exposure, combat exposure was most common among veterans with service dogs. Veterans with service dogs were significantly more likely to indicate combat exposure than those with companion dogs (*p* < 0.05). Other differences among dog functional groups did not reach statistical significance.

There were significant differences in mean psycho-social health scores of veterans with the three dog types (see Table 2). Scores differed for four of the outcomes: PTSD symptom severity, anxiety, physical well-being, and suicidal ideation. There was also a tendency for depression to differ among veterans with the three functional dog types. In terms of PTSD, for all veterans, symptom severity varied widely. However, the overall mean of 55.08 (*SD* = 21.6) is well above the cutoff of 33 or higher, indicating probable PTSD in this sample of veterans. PTSD symptom severity differed significantly among the functional dog types [*F*(2,237) = 6.34]. PTSD symptoms were less severe among veterans with CDs than those with SDs (*p* = 0.002) or ESDs (*p* = 0.035). Anxiety was less severe among veterans with CDs than those with SDs (*p* = 0.012), but anxiety among veterans with ESDs was intermediate and did not differ significantly from either of the other groups. Physical well-being was significantly lower for veterans with SDs than those with CDs (*p* = 0.05) or ESDs (*p* = 0.035). Positive affect, loneliness, stress, mental well-being and companionship did not differ significantly among veterans with the three functional dog types (see Table 2).

### 3.4. Dog Relationship Differences by Functional Dog Type

Overall, dog relationships, as assessed via the CDORS and the three dog relationship subscales (interaction, closeness, and cost comparisons), differed significantly by dog functional type (see Table 3). In all cases except closeness, which did not differ, post hoc analyses revealed that relationships with SDs were significantly stronger than relationships with ESDs (CDORS: Total: *p* < 0.001; CDORS interaction: *p* = 0.002; CDORS closeness: *p* = 0.261; cost: *p* = 0.001). In all cases, relationships with SDs were stronger than those with CDs (CDORS Total: *p* < 0.001; CDORS interaction: *p* < 0.001; CDORS closeness: *p* = 0.001; CDORS cost: *p* = 0.007). The strength of veterans’ relationships with their CDs did not differ significantly from the strength of those with their ESDs (CDORS Total: *p* = 0.20; CDORS interaction: *p* = 0.086; CDORS closeness: *p* = 0.182; CDORS cost: *p* = 1.00).

### 3.5. Dog Relationship Correlations with Health Characteristics

In examining the entire sample, the strength of the veteran’s overall dog relationship was correlated with greater PTSD symptom severity, anxiety, depression, loneliness, perceived stress, and lower physical and mental well-being (see Table 4). Dog relationship strength was not related to positive effect or suicidal ideation. When the subscales were examined separately, among the entire sample, higher interaction scores (CDORS sub-scales) were associated with lower physical well-being and lower suicidal ideation. Higher closeness scores were related to lower suicidal ideation and higher companionship, while higher dog cost was related to greater positive affect, lower suicidal ideation and greater companionship.

When the three functional dog types were considered separately, a stronger overall dog relationship was correlated significantly with higher PTSD symptoms and loneliness and lower physical well-being among veterans with SDs, but not among veterans with ESDs or CDs. A stronger dog relationship was also correlated with higher perceived stress and companionship among veterans with CDs, but not among veterans with other functional dog types.

When the relationships of the subscales to the psycho-social outcomes in veterans with the dog types were examined separately, there were few significant correlations. The exceptions were emotional closeness with the dog for those who owned SDs and suicidal ideation among veterans with all three functional dog types.

Higher perceived emotional closeness (CDORS sub-scale) with their dogs was related to lower anxiety, depression, loneliness, and perceived stress among veterans with SDs, but not other types of dogs. Higher dog interaction was associated with lower suicidal ideation in veterans with CDs and SDs, but not ESDs. Higher dog closeness and higher dog cost were also associated with lower suicidal ideation among veterans with all three dog types. In addition, higher perceived stress was associated with higher dog interaction scores among veterans with CDs, but not ESDs or SDs. Higher dog closeness and dog cost were associated with higher companionship only among veterans with SDs.

## 4. Discussion

### 4.1. Overview

This study is the first to examine the relationship among demographics and health characteristics and intensity of the human–dog relationship in veterans who cohabite with SDs, ESDs, or CDs. Despite the fact that veterans are relying on their dogs for a multitude of physical and mental health issues, in the literature, there has been a clear lack of evidence on which functional dog type is the most appropriate, based on veteran characteristics. This is an important factor when clinicians are discussing rehabilitative interventions with the veterans who serve.

### 4.2. Mental Health Needs and the Functional Dog Types

Based on the results of this study, it appears that veterans who need more assistance with mental health challenges own specially trained SDs. For example, veterans with an SD reported experiencing combat exposure (56.8%), compared to veterans with CDs (31.7%) or ESDs (48.1%). Veterans with SDs also had more mental health challenges (i.e., higher symptoms of PTSD and anxiety and lower physical well-being) compared to veterans with ESDs or CDs. Moreover, research indicates that SDs and CDs are associated with improvements in PTSD symptoms (Leighton et al., 2022; Ortmeyer & Robey, 2019; Stern et al., 2013; Stern et al., 2022).

### 4.3. Gender Differences Among Service Dog Owners

An interesting finding generated by this research was that more women veterans owned SDs compared to men. This finding is in line with existing literature suggesting that women are more likely to seek treatment for mental health issues than men in the civilian population (Pinkhasov et al., 2010). Within the military population, it is known that male service members often underutilize mental health services (Hom et al., 2017). Further exploration is suggested to identify the underlying factors hindering men from seeking support from an SD.

### 4.4. Human–Dog Interaction as a Source of Social and Mental Health Support

Social interaction has been shown to influence health, including biological, psychological, and social outcomes (Umberson & Karas Montez, 2010). Supportive social ties can also create norms that influence healthy behaviors (Umberson & Karas Montez, 2010). Interacting with others (e.g., humans and dogs) can support mental health by providing a form of emotional and social support (Krause-Parello, 2012). Social support is also considered a protective factor for suicide (Pietrzak et al., 2017). Dogs may also provide veterans with a sense of purpose and a reason to get up in the morning due to the caretaking responsibilities of dog ownership. In this study, higher dog interaction was associated with lower suicidal ideation among SD and CD owners, and perceived emotional closeness was associated with lower suicidal ideation among all functional dog ownership groups. Since suicide among veterans is considered a public health concern (Sareen et al., 2016; U.S. Department of Veterans Affairs Office of Mental Health and Suicide Prevention, 2016), and is associated with PTSD, these findings support that having a dog may be therapeutic to veterans who are at risk for suicide.

### 4.5. Human–Dog Relationship Intensity and Mental Health

The intensity of the veteran’s relationship with their dog, as measured by the overall CDORS, was associated with suboptimal mental health, specifically higher PTSD symptoms, anxiety, depression, loneliness, perceived stress, and lower physical and mental well-being. Veterans in this study who experienced mental health challenges felt close to their dogs, perhaps as a source of comfort, as reflected in the CDORS closeness score.

### 4.6. Connection Between Emotional Closeness/Interaction and Rehabilitative Health Benefits

In this study, veterans who had a high degree of emotional closeness and interaction with their SD may also have experienced more rehabilitative health benefits. For example, higher emotional closeness among veterans with SDs was associated with lower anxiety, depression, loneliness, and perceived stress. These findings suggest that rehabilitation interventions should consider ways to increase closeness and interaction among veterans and their dogs.

### 4.7. Suggestions for Future Research

Although this research filled a fundamental gap in the literature, there are still unknown factors, such as what functional dog type is the most cost-effective and under what circumstances, and how much dog exposure targets health and well-being outcomes. We acknowledge that differences in breed types may have impacted the results; however, accounting for breed variation was beyond the scope of this study. Future research should be conducted to examine these factors. Nonetheless, comparing the three functional dog types remained an important and valid objective to better understand their distinct impacts on veteran outcomes. Charged with this type of information, clinicians and veterans alike can select the functional dog type that will maximize health and rehabilitation efforts for the least cost.

### 4.8. Study Limitations

There are limitations to this cross-sectional study. In this study, we used convenience sampling. While the online nature of this study allowed veterans from many different locations to participate, it also created a limitation in that those without internet access were not able to participate. This, along with the non-probability sampling method, may result in conclusions that are less generalizable to the veteran demographic as a whole. We also recognize that the VR-12 subscales, the MCS and PCS, demonstrated low reliability and therefore should be interpreted with caution. These subscales were scored with the SAS 9.4M8 program syntax provided by Boston University, the developers of the measure. Based on these scores, the veterans who participated in this project had low well-being on average. A thorough literature search did not reveal the reliability of the VR-12 measure, but this measure is highly correlated with the MOS Short-form Health Survey version 2 (SF-12v2), which is well validated (Fong et al., 2022).

## 5. Conclusions

This study provides insight into the potential role dogs may play in improving mental health and well-being in veterans. Clinicians should consider using the results of this study to make better-informed decisions and incorporate dogs in personalized care plans for the improvement of mental health and well-being, ensuring the right functional dog type is matched with the specific needs of the veteran.

## Figures and Tables

**Table 1:** Demographic characteristics of the veterans and their dogs according to dog functional type.

Characteristic	All (*N* = 242)		Companion (*N* = 81)		Emotional Support (*N* = 80)		Service Dog (*N* = 81)			
	Mean	*SD*	Mean	*SD*	Mean	*SD*	Mean	*SD*	*F* (*df*)	*p*
Veteran Age	46.9	13.4	47.9	13.2	46.3	12.6	47.8	12.6	0.40 (2,232)	0.671
Dog Age	5.51	3.61	6.5	4.5	5.0	3.4	5.0	2.5	4.46 (2,222)	0.013
Characteristic	All (*N* = 242)		Companion (*N* = 81)		Emotional Support (*N* = 80)		Service Dog (*N* = 81)			
	*N* Value	%	*N* Value	%	*N* Value	%	*N* Value	%	*Χ2* (*df*)	*p* Value
Gender Identity										
Male	143	59.6	40	50	29	36.7	26	32.1	7.12 (4)	0.13
Female	95	39.6	39	48.8	49	62	55	67.9		
Another identity not listed	2	0.8	1	1.3	1	1.3	0	0		
Race/Ethnicity										
White	172	71.1	59	72.8	51	63.7	62	76.5	3.39 (2)	0.184
Black	31	12.8	10	12.3	14	17.5	7	8.6	2.85 (2)	0.24
Hispanic or Latino	18	7.4	6	7.4	6	7.5	6	7.4	0.00 (2)	1.0
Middle Eastern	1	0.4	0	0	0	0	1	1.2	2.00 (2)	0.369
Native American	12	5.0	4	4.9	4	5	4	4.9	0.00 (2)	1.0
Pacific Islander	4	1.7	1	1.2	1	1.3	2	2.5	0.50 (2)	0.779
Other	5	2.1	1	1.2	1	1.3	3	3.7	1.61 (2)	0.446
Decline to State	3	1.2	2	2.5	1	1.3	0	0	2.02 (2)	0.365
Education										
HS grad or less	13	5.8	3	3.7	7	8.8	4	4.9	11.13 (8)	0.195
Some College or Associate’s Degree	94	38.8	34	42	28	35	32	39.5		
Bachelor’s Degree	57	23.6	24	29.6	20	25	13	16		
Graduate Degree	77	31.8	20	24.7	25	31.3	32	39.5		
Marital Status										
Married/Domestic Partnership	140	58.1	43	53.1	54	68.4	43	53.1	10.87 (10)	0.368
Divorced	62	25.7	20	24.7	17	21.5	25	30.9		
Separated	3	1.2	1	1.2	0	0	2	2.5		
Widowed	5	2.1	2	2.5	1	1.3	2	2.5		
Single (never married)	31	12.9	15	18.5	7	8.9	9	11.1		
Has Children										
Yes	153	63.5	50	61	48	60.8	55	68.8	1.44 (2)	0.486
No	88	36.5	32	39	31	39.2	25	31.3		
Employment Status										
Working Full Time	88	36.4	34	41.4	29	36.7	25	30.9	24.86 (18)	0.129
Working Part Time	10	4.1	3	3.6	2	2.6	5	6.1		
Working Sporadically	3	1.2	0	0	3	3.8	0	0		
Unemployed (Looking for Work)	4	1.7	1	1.2	3	3.8	0	0		
In School Full/Part Time	19	7.9	6	7.3	10	12.7	3	3.7		
Full Time Caregiver	8	3.3	4	4.9	3	3.8	1	1.2		
Retired	44	18.2	13	15.9	13	16.5	18	22.2		
Disabled	66	27.3	21	25.6	16	20.3	29	35.8		
Living Arrangements										
Rent	64	26.4	18	22	24	30.4	22	27.2	6.44 (8)	0.598
Own a Residence	158	65.3	59	72	48	60.8	51	63		
Reside with Friends/Family	10	4.1	2	2.4	3	3.8	5	6.2		
Homeless	3	1.2	1	1.2	2	2.5	0	0		
Other	7	2.9	2	2.4	2	2.5	3	3.7		
Owned a Dog as A Child										
Yes	182	75.2	65	79.3	52	65.8	65	80.2	5.18 (2)	0.75
No	60	24.8	17	20.7	27	34.2	16	19.8		
Combat Exposure										
Yes	110	45.6	26	31.7	38	48.1	46	56.8	11.63 (2)	0.003
No	131	54.4	56	68.3	40	50.6	35	43.2		
Military Branch										
Air Force	47	19.4	19	23.5	13	16.3	15	18.5	1.40 (2)	0.497
Army	117	48.3	32	39.5	46	57.5	39	48.1	5.22 (2)	0.074
Navy	41	16.9	15	18.5	10	12.5	16	19.8	1.72 (2)	0.423
Marines	34	14.0	11	13.6	10	12.5	13	16	0.44 (2)	0.802
Coast Guard	7	2.9	3	3.7	3	3.8	1	1.2	1.19 (2)	0.551
National Guard	17	7.0	5	6.2	5	6.3	7	8.6	0.49 (2)	0.784

**Table 2:** Health of Veteran Dog Owners: Comparison of Veterans who have Dogs in Each Functional Group.

Characteristic	All *N* = 242		Companion Dog *N* = 81		Emotional Support Dog *N* = 80		Service Dog *N* = 81		ANOVA	
	Mean	*SD*	Mean	*SD*	Mean	*SD*	Mean	*SD*	*F* (*df*)	*p* Value
PTSD Symptoms	55.08	21.57	38.44	20.11	56.94	22.54	59.9	20.55	6.34 (2,237)	0.002
Anxiety	60.90	8.88	58.77	8.51	61.10	9.01	62.89	8.72	4.46 (2,239)	0.013
Positive Affect	37.96	10.29	39.28	10.44	38.13	10.19	36.45	10.17	1.53 (2,238)	0.218
Depression	10.67	6.39	9.51	6.65	10.60	6.24	11.94	6.12	2.95 (2,239)	0.055
Loneliness	19.51	5.72	18.64	5.96	19.61	5.43	20.29	5.70	1.69 (2,237)	0.187
Stress	22.96	4.20	22.59	4.24	22.57	4.49	23.73	3.76	2.02 (2,239)	0.135
Physical Well-being	39.23	11.88	40.65	12.96	40.89	10.85	36.19	11.24	3.88 (2,239)	0.022
Mental Well-being	38.62	13.45	40.26	14.17	38.25	12.45	37.32	13.65	1.14 (2,239)	0.322
Suicidal Ideation	6.3	9.34	4.65	7.83	6.3	9.71	8.03	10.16	3.08 (2,237)	0.048
Companionship	45.75	9.99	45.46	10.14	45.58	9.37	46.23	10.57	0.14 (2,238)	0.873

For specific scale names, refer to line 129.

**Table 3:** Veterans’ Relationships with their Dogs (CDORS Total and CDORS Interaction/Closeness/Cost): Comparison of Veterans with Dogs in Each Functional Group.

CDORS Scores	All *N* = 242		Companion Dog *N* = 81		Emotional Support Dog *N* = 80		Service Dog *N* = 81		ANOVA	
	Mean	*SD*	Mean	*SD*	Mean	*SD*	Mean	*SD*	*F* (*df*)	*p* Value
Total	137.33	14.33	132.02	14.04	135.95	13.81	144.11	12.47	15.93 (2,238)	<0.001
Interaction	4.17	0.57	3.95	0.59	4.14	0.57	4.43	0.45	15.87 (2,238)	<0.001
Closeness	4.09	0.62	4.21	0.64	4.39	0.56	4.55	0.63	6.39 (2,238)	0.002
Cost	4.09	0.70	3.95	0.74	3.96	0.73	4.35	0.57	7.45 (2,239)	<0.001

Note. Ns for analyses vary slightly due to missing data on CDORSs.

**Table 4:** Correlations of Veterans’ Health Outcomes with Dog Relationship for all Veterans and Veterans with Each Functional Type of Dog.

	All Participants (*N* = 242)	Companion Dog Owners (*N* = 81)	Emotional Support Dog Owners (*N* = 80)	Service Dog Owners (*N* = 81)
CDORS Total				
PTSD Symptoms	0.223 **	0.198	0.162	0.272 *
Anxiety	0.134 *	0.133	0.125	0.145
Positive Affect	−0.059	0.096	−0.067	−0.272 **
Depression	0.158 **	0.146	0.098	0.159
Loneliness	0.223 **	0.059	0.147	0.451 **
Perceived Stress	0.268 **	0.303 **	0.16	0.174
Physical Well-being	−0.206 **	−0.170	−0.09	−0.323 **
Mental Well-being	−0.124 *	−0.129	−0.025	−0.12
Suicidal Ideation	−0.044	−0.131	0.012	−0.005
Companionship	0.072 *	0.217 *	0.06	−0.062
CDORS Interaction				
PTSD Symptoms	0.087	0.026	0.013	0.008
Anxiety	0.74	0.060	0.009	−0.059
Positive Affect	−0.08	−0.141	−0.021	0.073
Depression	0.087	0.115	0.018	−0.073
Loneliness	0.055	0.109	0.02	−0.121
Perceived Stress	0.097	0.247 *	0.002	−0.109
Physical Well-being	−0.194 **	−0.137	−0.209	−0.08
Mental Well-being	−0.062	−0.091	−0.035	0.054
Suicidal Ideation	−0.178 **	−0.33 **	−0.20	−0.247 *
Companionship	0.093	0.014	0.095	0.173
CDORS Closeness				
PTSD Symptoms	0.007	−0.040	0.042	−0.181
Anxiety	−0.006	−0.016	0.044	−0.242 *
Positive Affect	0.074	0.034	0.110	0.221
Depression	−0.022	0.000	−0.001	−0.258 *
Loneliness	−0.021	−0.024	0.050	−0.223 *
Perceived Stress	−0.030	−0.023	0.034	−0.276 *
Physical Well-being	−0.087	−0.040	−0.068	−0.030
Mental Well-being	−0.013	0.042	−0.105	0.098
Suicidal Ideation	−0.269 **	−0.377 **	−0.233 *	−0.379 **
Companionship	0.125 *	−0.016	0.086	0.352 **
CDORS Dog Cost				
PTSD Symptoms	−0.029	−0.133	−0.056	−0.040
Anxiety	−0.053	−0.055	−0.138	−0.108
Positive Affect	0.130 *	0.179	0.178	0.137
Depression	−0.075	−0.063	−0.155	−0.150
Loneliness	−0.088	−0.105	−0.119	−0.139
Perceived Stress	−0.026	0.147	−0.160	−0.175
Physical Well-being	−0.044	0.025	−0.027	−0.002
Mental Well-being	0.040	0.070	0.049	0.072
Suicidal Ideation	−0.236 **	−0.374 **	−0.259 *	−0.234 *
Companionship	0.201 **	0.198	0.151	0.252 *

* Correlation is significant at the 0.05 level (2-tailed). ** Correlation is significant at the 0.01 level (2-tailed). For specific scale names, refer to line 129.

## Data Availability

The datasets presented in this article are not readily available because the data is still being used for other parts of the research, so releasing it now would interfere with that work.
